# Risk factors for surgical site infection following spinal surgery: A meta-analysis

**DOI:** 10.1097/MD.0000000000028836

**Published:** 2022-02-25

**Authors:** Xinxin Zhang, Peng Liu, Jipeng You

**Affiliations:** a Emergency Department, Affiliated Hospital of Hebei University, No. 212 Road Yuhua Dong, Baoding, Hebei, China; b Medical Department, Affiliated Hospital of Hebei University, No. 212 Road Yuhua Dong, Baoding, Hebei, China.

**Keywords:** incidence, meta-analysis, risk factors, spinal surgery, surgical site infection

## Abstract

**Study design:**

A meta-analysis.

**Background:**

We performed a meta-analysis to explore risk factors of surgical site infection (SSI) following spinal surgery.

**Methods:**

An extensive search of literature was performed in English database of PubMed, Embase, and Cochrane Library and Chinese database of CNKI and WANFANG (up to October 2020). We collected factors including demographic data and surgical factor. Data analysis was conducted with RevMan 5.3 and STATA 12.0.

**Results:**

Totally, 26 studies were included in the final analysis. In our study, the rate of SSI after spinal surgery was 2.9% (1222 of 41,624). Our data also showed that fusion approach (anterior vs posterior; anterior vs combined), osteotomy, transfusion, a history of diabetes and surgery, hypertension, surgical location (cervical vs thoracic; lumbar vs thoracic), osteoporosis and the number of fusion levels were associated with SSI after spinal surgery. However, age, sex, a history of smoking, body mass index, fusion approach (posterior vs combined), surgical location (cervical vs lumbar), duration of surgery, blood loss, using steroid, dural tear and albumin were not associated with development of SSI.

**Conclusions:**

In our study, many factors were associated with increased risk of SSI after spinal surgery. We hope this article can provide a reference for spinal surgeons to prevent SSI after spinal surgery.

## 1 Introduction

Surgical site infection (SSI), as the third most common complication, always brings in miserable and poor outcomes.^[1–3]^ Previous studies^[4–6]^ have been reported 0.2% to 16.1% occurrence of patients who underwent spinal surgery. Undoubtedly, SSI not only prolong hospital stay for the patients, but also increase medical, social, and economical costs. Thus, it is important to find the risk factors for SSI to lower rate of infection after spinal surgery.

A variety of risk factors including diabetes, obesity, longer operation times, smoking, history of previous SSI, type of surgical approach, larger blood loss, and use of spinal instrumentation surgery have been mentioned by previous studies.^[7,8]^ As we know, many studies have reported the incidence and risk factors of SSI following spinal surgery, yet previous meta-analysis has just studied the epidemiological incidence of SSI after spinal surgery. To our knowledge, there is few meta analysis regarding risk factors of SSI after spinal surgery. Therefore, this study aims to explore incidence and risk factors of SSI following spinal surgery.

## 2 Methods

### 2.1 Statement of ethics

This study was approved by the institutional review board (IRB) of our hospital. An informed consent from the patients was not considered necessary by the Ethics Committee as our data originated from published papers. The present study has been conducted ethically in accordance with the World Medical Association Declaration of Helsinki.

### 2.2 Search strategy

We searched for the English and Chinese language studies with the keywords: “surgical site infection” or “SSI”, and “spinal surgery” in English database of PubMed, Embase, and Cochrane Library and Chinese database of CNKI and WANFANG. There was no limitation on the date of publication, which covered all previously published studies up to October 2020.

### 2.3 Eligibility criteria

Included articles must satisfy: study population must be adult patients (>18 years old); measured out comes of the incidence and risk factors of SSI after spine surgery; comparison: SSI group and non-SSI group; Studies were excluded if they were abstracts, letters, reviews or case reports; had repeated data; did not report outcomes of interest.

### 2.4 Data extraction and outcome measures

The data included the general characteristics of each study and the outcomes measured. General characteristics included first author, year of publication, country, the number of SSI patients and total patients, type of article, shown in Table 1. When the same population was reported in several publications, we retained only the most informative article or complete work to avoid duplication of information. Data were extracted independently by 2 authors. Any disagreements concerning paper eligibility were resolved by discussion and consensus. Test for risk of publication bias. We performed a visual inspection of the funnel plot for publication bias. The funnel plot should be asymmetric when there is publication bias and symmetric in the case of no publication bias. We performed Egger and Begg tests to measure the funnel plot asymmetry using a significance level of *P* < .10. The trim and fill computation was used to estimate the effect of publication bias. Sensitive analysis overall because of the low heterogeneity of every factor, so we do not calculate sensitive analysis.

**Table 1:** Characteristics of included studies.

			No. of participants		
First author	Year	Country	ASD	Total	Study type
Amy M. Cizik^[10]^	2012	USA	63	1532	Retrospective
Andrew A. Fanous^[11]^	2019	USA	20	532	Retrospective
CJ. Lucasti^[12]^	2019	USA	13	74	Retrospective
Cindy R. Nahhas^[13]^	2017	USA	108	2548	Retrospective
John J. Lee^[14]^	2016	USA	15	149	Retrospective
Kotaro Satake^[15]^	2013	USA	11	110	Retrospective
Qi Lai^[16]^	2017	China	26	923	Retrospective
Satoshi Ogihara^[17]^	2015	Japan	24	2736	Retrospective
Satoshi Ogihara^[18]^	2018	Japan	26	4027	Retrospective
Satoshi Ogihara^[19]^	2019	Japan	20	623	Retrospective
Sjoerd P. F. T. Nota^[20]^	2015	USA	361	5761	Retrospective
Takashi Sono^[21]^	2018	Japan	10	637	Retrospective
Samer Habiba^[22]^	2017	Norway	40	1772	Retrospective
SHI Lei^[23]^	2017	China	36	3964	Retrospective
Oren G. Blam^[24]^	2003	USA	24	256	Retrospective
Nathan J. Lee^[25]^	2017	USA	140	5803	Retrospective
Muneharu Ando^[26]^	2014	Japan	8	294	Retrospective
Matt El-Kadi^[27]^	2019	USA	30	5065	Retrospective
Jin-Sol Han^[28]^	2016	Korea	10	280	Retrospective
Albert F^[29]^	2010	USA	46	830	Retrospective
Daniël M. C. Janssen^[30]^	2018	Netherlands	60	898	Retrospective
Eiichiro Iwata^[31]^	2016	Japan	5	85	Retrospective
Jin Hak Kim^[32]^	2015	Korea	30	1831	Retrospective
Yusuke Yamamoto^[33]^	2018	Japan	11	141	Retrospective
Can Yaldiz^[34]^	2015	Turkey	63	540	Retrospective
Ankit I. Mehta^[35]^	2013	USA	22	213	Retrospective

### 2.5 Statistical analysis

Dichotomous outcomes were presented as odd ratios (OR) and 95% confidence intervals (CI) were calculated for outcomes, while continuous variable were regarded as standardized mean difference (SMD) and 95% CI. A *P* value < .05 was judged as statistically significant. Random-effects or fixed-effects models were used depended on the heterogeneity of the studies included. Heterogeneity was analyzed with both the Chi squared test *I* square test, where *P* value of < .10 for the Chi squared and *I*^2^ > 50% implied heterogeneity.^[9]^ All statistical analyses were performed using Review Manager version 5.3 (The Cochrane Collaboration, Oxford, UK) and STATA 12.0 (Stata Corporation, College Station, TX).

## 3 Results

### 3.1 Study identification and selection

**Figure 1:**
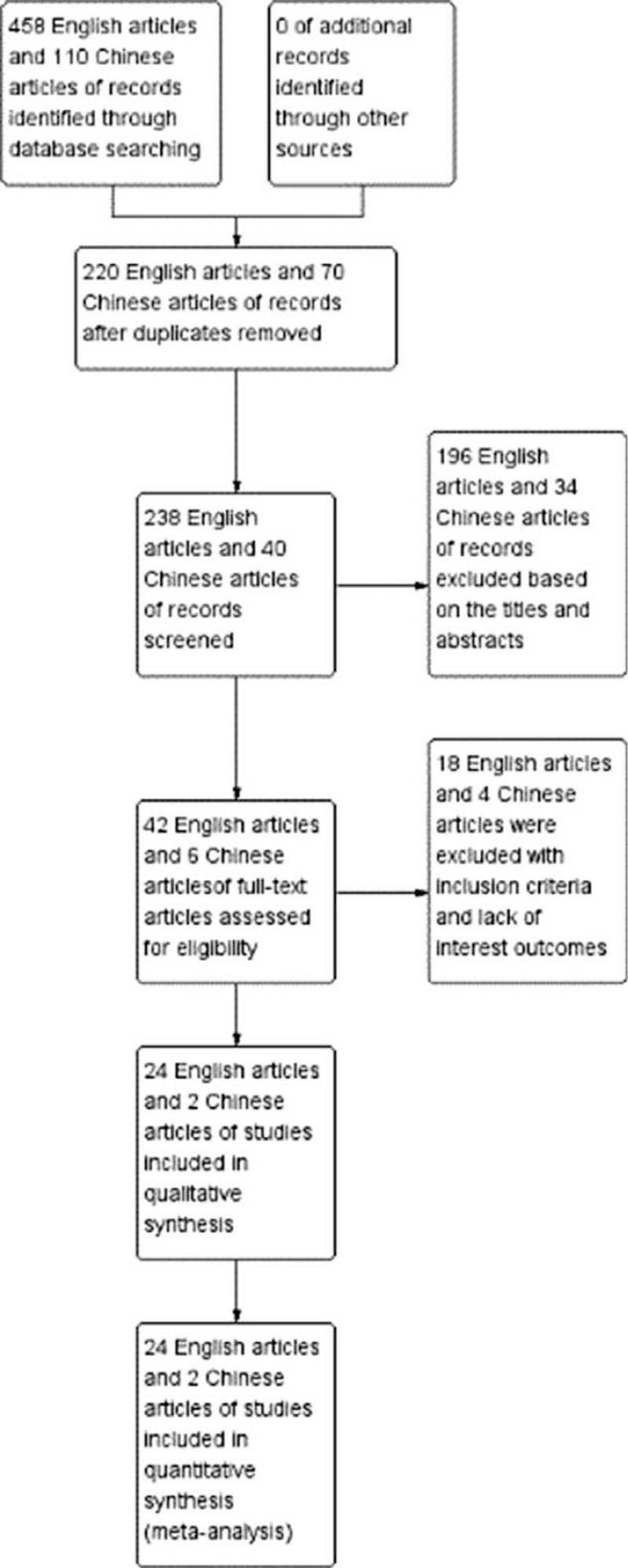
Flow diagram of study selection.

Initially, we collected totally 568 (458 English articles and 110 Chinese articles) records by the database search. Two hundred ninety records (220 English articles and 70 Chinese articles) were excluded due to repetition and 230 (196 English articles and 34 Chinese articles) records were removed for review based on the titles and abstracts. The remaining 48 records were retrieved for inclusion criteria and 15 (13 English articles and 2 Chinese articles) of them were excluded, 7 (5 English articles and 2 Chinese articles) did not report outcomes of interest. Finally, 26 (24 English articles and 2 Chinese articles) articles that met our inclusion criteria were included in the present meta-analysis. The selection process that included in this meta-analysis is shown in Figure 1.

### 3.2 Baseline characteristics and quality assessment

The main characteristics of the 26 articles (from 74 to 5803 patients) that published before October 2020 included in the meta-analysis were presented in Table 1. Finally, 1222 patients were suffering from SSI after spinal surgery in total of 41,264 patients. According to the 26 included studies, the rate of SSI was 2.9% (ranged from 0.6%-17.6%).

Because all studies included were retrospective studies, we used the Newcastle Ottawa Quality Assessment Scale (NOQAS) to assess the quality of each study. This scale for non-randomized case controlled studies and cohort studies were used to allocate a maximum of 9 points for the quality of selection, comparability, exposure, and outcomes for study participants. Of these studies, 19 studies scored 8 points and 7 studies scored 7 points. Hence, the quality of each study was relatively high (Table 2).

**Table 2:** The quality assessment according to the Newcastle Ottawa Quality Assessment Scale (NOQAS) of each study.

Study	Selection	Comparability	Exposure	Total score
Amy M. Cizik^[10]^	3	3	2	8
Andrew A. Fanous^[11]^	3	2	3	8
CJ. Lucasti^[12]^	2	3	3	8
Cindy R. Nahhas^[13]^	2	3	2	7
John J. Lee^[14]^	3	3	2	8
Kotaro Satake^[15]^	3	2	2	7
Qi Lai^[16]^	3	3	2	8
Satoshi Ogihara^[17]^	2	3	3	8
Satoshi Ogihara^[18]^	3	3	2	8
Satoshi Ogihara^[19]^	3	2	3	8
Sjoerd P. F. T. Nota^[20]^	2	2	3	7
Takashi Sono^[21]^	3	2	3	8
Samer Habiba^[22]^	2	3	3	8
SHI Lei^[23]^	2	3	2	7
Oren G. Blam^[24]^	3	3	2	8
Nathan J. Lee^[25]^	3	2	2	7
Muneharu Ando^[26]^	3	3	2	8
Matt El-Kadi^[27]^	3	3	2	8
Jin-Sol Han^[28]^	2	3	3	8
Albert F^[29]^	3	2	2	7
Daniël M. C. Janssen^[30]^	3	2	3	8
Eiichiro Iwata^[31]^	2	3	3	8
Jin Hak Kim^[32]^	2	3	3	8
Yusuke Yamamoto^[33]^	3	2	2	7
Can Yaldiz^[34]^	3	3	2	8
Ankit I. Mehta^[35]^	3	2	3	8

### 3.3 Assessment of risk factors of SSI

#### 3.3.1 Age

**Figure 2:**
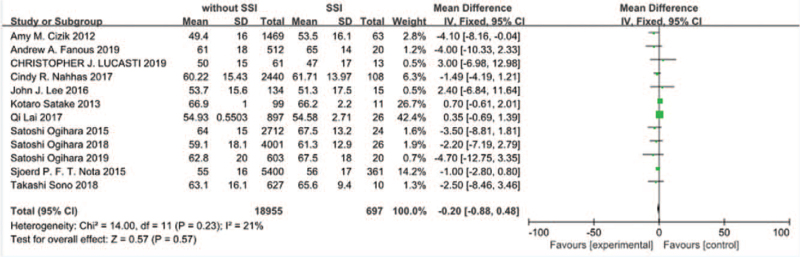
The standardized mean difference (SMD) estimate preoperative age in 2 groups.

Twelve studies^[10–21]^ reported age of patients at operational time between SSI group and non-SSI group. There was not significant in the test for heterogeneity and the studies had low heterogeneity (*P* for heterogeneity = .23; *I*^2^ = 21%, Fig. 2). The meta-analysis showed that age was not associated with a significant increase in the incidence of SSI (fixed-effects model; *P* = .57, SMD = –0.20, 95% CI [–0.88, 0.48], Fig. 2).

#### 3.3.2 Body mass index (BMI)

**Figure 3:**
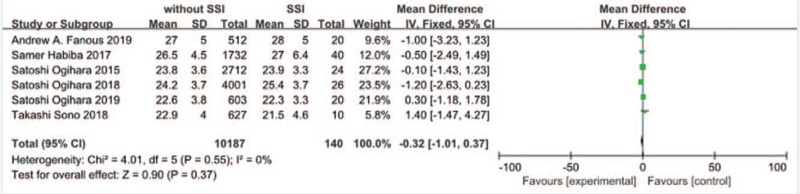
The standardized mean difference (SMD) estimate preoperative body mass index in 2 groups.

Six studies^[11,17–19,21,22]^ reported BMI of patients at operational time between SSI group and non-SSI group. There was not significant in the test for heterogeneity and the studies had low heterogeneity (*P* for heterogeneity = .55; *I*^2^ = 0%, Fig. 3). The meta-analysis showed that BMI was not associated with a significant increase in the incidence of SSI (fixed-effects model; *P* = .37, SMD = –0.32, 95% CI [–1.01, 0.37], Fig. 3).

#### 3.3.3 Smoking

**Figure 4:**
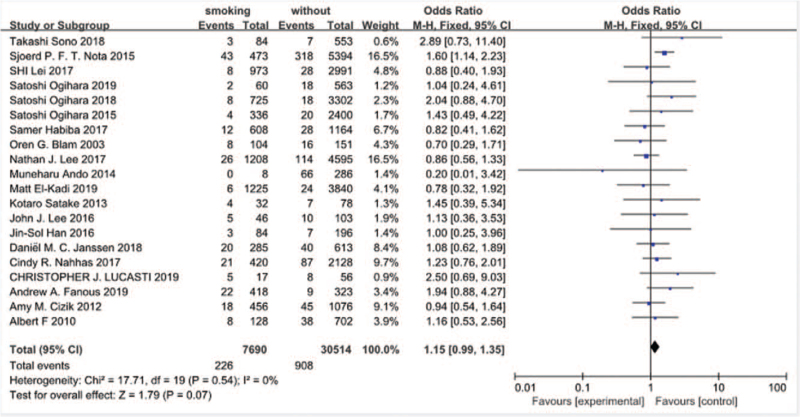
The odds ratio (OR) estimate for history of smoking.

Twenty studies^[10–15,17–29]^ reported a history of smoking between SSI group and non-SSI group. There was not significant in the test for heterogeneity and the studies had low heterogeneity (*P* for heterogeneity = .54; *I*^2^ = 0%, Fig. 4). The meta-analysis showed that history of smoking was not associated with a significant increase in the incidence of SSI (fixed-effects model; *P* = .07, OR = 1.15, 95% CI [0.99, 1.35], Fig. 4).

#### 3.3.4 Sex

**Figure 5:**
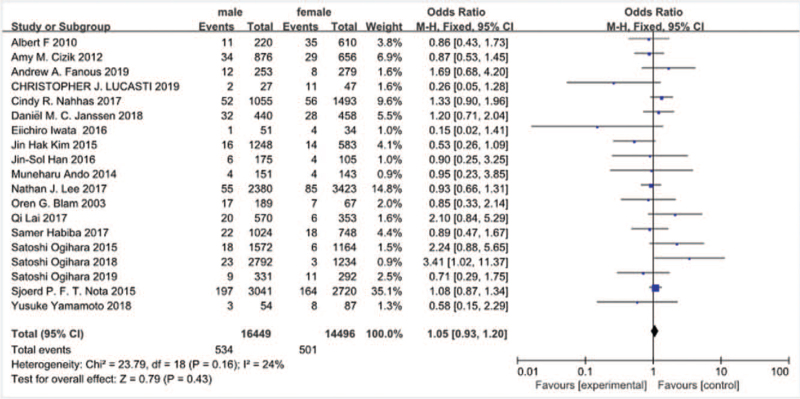
The odds ratio (OR) estimate for gender.

Nineteen studies^[10–13,16–20,24–26,28–33]^ reported sex between SSI group and non-SSI group. There was not significant in the test for heterogeneity and the studies had low heterogeneity (*P* for heterogeneity = 0.16; *I*^2^ = 24%, Fig. 5). The meta-analysis showed that gender was not associated with a significant increase in the incidence of SSI (fixed-effects model; *P* = .43, OR = 1.05, 95% CI [0.93, 1.20], Fig. 5).

#### 3.3.5 Diabetes

**Figure 6:**
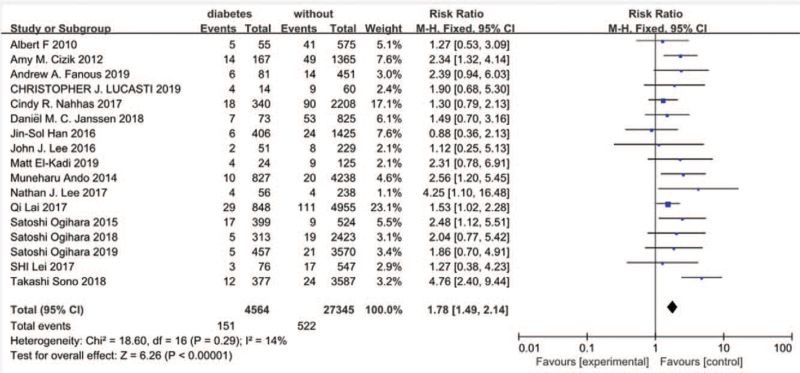
The odds ratio (OR) estimate for history of diabetes.

Seventeen studies^[10–14,16–19,21,23,25–30]^ reported a history of diabetes between SSI group and non-SSI group. There was not significant in the test for heterogeneity and the studies had low heterogeneity (*P* for heterogeneity = .29; *I*^2^ = 14%, Fig. 6). The meta-analysis showed that a history of diabetes was associated with a significant increase in the incidence of SSI (fixed-effects model; *P* < .00001, OR = 1.78, 95% CI [1.49, 2.14], Fig. 6).

#### 3.3.6 Hypertension

**Figure 7:**
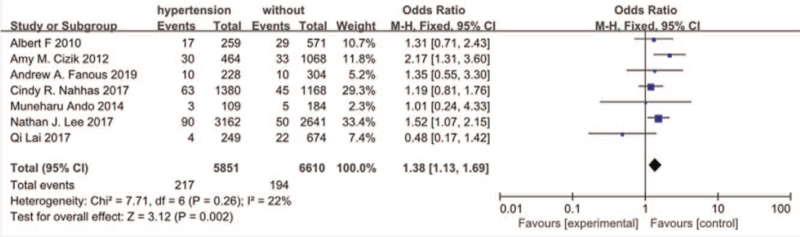
The odds ratio (OR) estimate for history of hypertension.

Seven studies^[10,11,13,16,25,26,29]^ reported a history of hypertension between SSI group and non-SSI group. There was not significant in the test for heterogeneity and the studies had low heterogeneity (*P* for heterogeneity = .26; *I*^2^ = 22%, Fig. 7). The meta-analysis showed that a history of hypertension was associated with a significant increase in the incidence of SSI (fixed-effects model; *P* = .002, OR = 1.38, 95% CI [1.13, 1.69], Fig. 7).

#### 3.3.7 Steroid use

**Figure 8:**
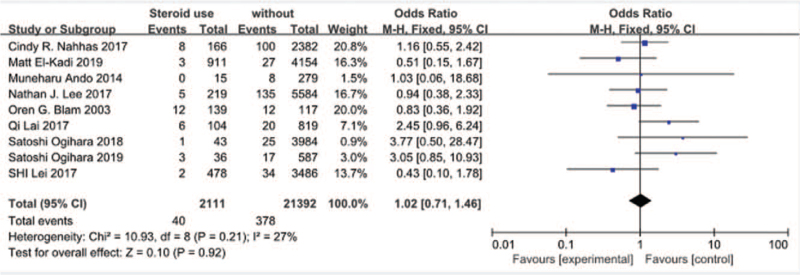
The odds ratio (OR) estimate for preoperative steroid use.

Nine studies^[13,16,18,19,23–27]^ reported steroid use between SSI group and non-SSI group. There was not significant in the test for heterogeneity and the studies had low heterogeneity (*P* for heterogeneity = .21; *I*^2^ = 27%, Fig. 8). The meta-analysis showed that steroid use not was associated with a significant increase in the incidence of SSI (fixed-effects model; *P* *=* *.92*, OR = 1.02, 95% CI [0.71, 1.46], Fig. 8).

#### 3.3.8 Osteoporosis

**Figure 9:**
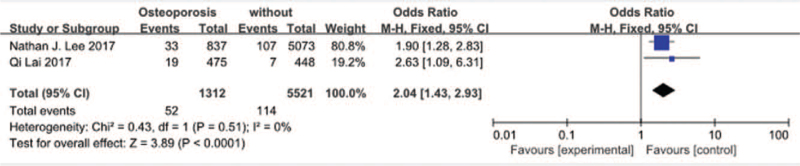
The odds ratio (OR) estimate for osteoporosis.

Two studies^[16,25]^ reported osteoporosis between SSI group and non-SSI group. There was not significant in the test for heterogeneity and the studies had low heterogeneity (*P* for heterogeneity = .51; *I*^2^ = 0%, Fig. 9). The meta-analysis showed that osteoporosis was associated with a significant increase in the incidence of SSI (fixed-effects model; *P* < *.0001*, OR = 2.04, 95% CI [1.43, 2.93], Fig. 9).

#### 3.3.9 Previous surgery

**Figure 10:**
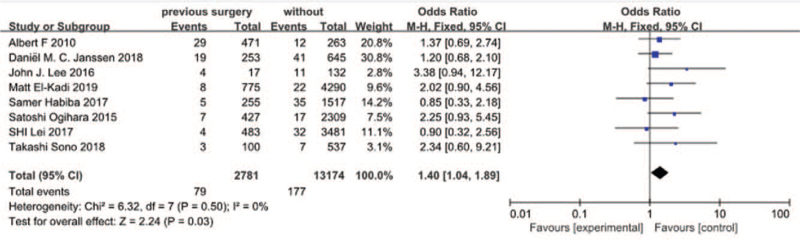
The odds ratio (OR) estimate for previous surgery.

Eight studies^[14,17,21–23,27,29,30]^ reported previous surgery between SSI group and non-SSI group. There was not significant in the test for heterogeneity and the studies had low heterogeneity (*P* for heterogeneity = .50; *I*^2^ = 0%, Fig. 10). The meta-analysis showed that previous surgery was associated with a significant increase in the incidence of SSI (fixed-effects model; *P* *=* *.03*, OR = 1.40, 95% CI [1.04, 1.89], Fig. 10).

#### 3.3.10 Albumin

**Figure 11:**
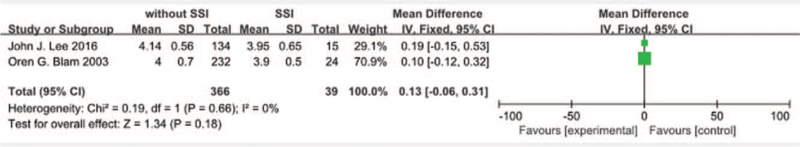
The standardized mean difference (SMD) estimate preoperative Albumin in 2 groups.

Two studies^[14,24]^ reported albumin of patients at operational time between SSI group and non-SSI group. There was not significant in the test for heterogeneity and the studies had low heterogeneity (*P* for heterogeneity = .66; *I*^2^ = 0%, Fig. 11). The meta-analysis showed that albumin was not associated with a significant increase in the incidence of SSI (fixed-effects model; *P* = .18, SMD = 0.13, 95% CI [–0.06, 0.331], Fig. 11).

#### 3.3.11 Osteotomy

**Figure 12:**
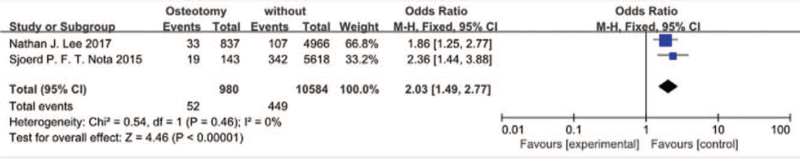
The odds ratio (OR) estimate for osteotomy.

Two studies^[20,25]^ reported osteotomy between SSI group and non-SSI group. There was not significant in the test for heterogeneity and the studies had low heterogeneity (*P* for heterogeneity = .46; *I*^2^ = 0%, Fig. 12). The meta-analysis showed that osteotomy was associated with a significant increase in the incidence of SSI (fixed-effects model; *P* < *.00001*, OR = 2.03, 95% CI [1.49, 2.77], Fig. 12).

#### 3.3.12 Transfusion

**Figure 13:**
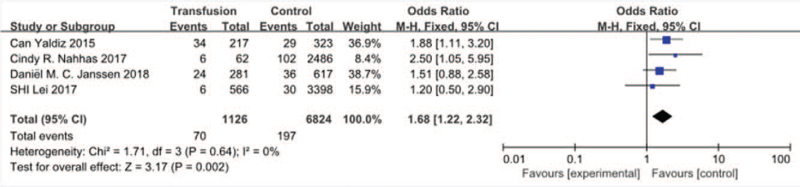
The odds ratio (OR) estimate for transfusion.

Four studies^[13,23,30,34]^ reported transfusion between SSI group and non-SSI group. There was not significant in the test for heterogeneity and the studies had low heterogeneity (*P* for heterogeneity = .64; *I*^2^ = 0%, Fig. 13). The meta-analysis showed that transfusion was associated with a significant increase in the incidence of SSI (fixed-effects model; *P* < *.002*, OR = 2.03, 95% CI [1.49, 2.77], Fig. 13).

#### 3.3.13 Dural tear

**Figure 14:**
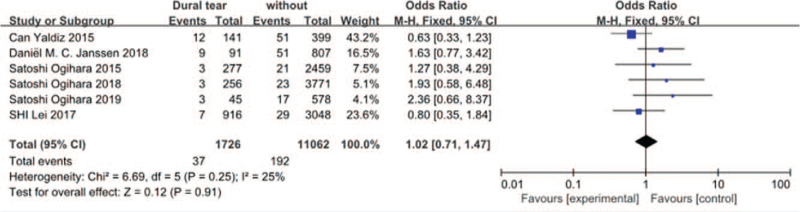
The odds ratio (OR) estimate for dural tear.

Six studies^[17–19,23,30,34]^ reported dural tear between SSI group and non-SSI group. There was not significant in the test for heterogeneity and the studies had low heterogeneity (*P* for heterogeneity = .25; *I*^*2*^ = 25%, Fig. 14). The meta-analysis showed that dural tear was not associated with a significant increase in the incidence of SSI (fixed-effects model; *P* *=* *.91*, OR = 1.02, 95% CI [0.71, 1.47], Fig. 14).

#### 3.3.14 Duration of surgery

**Figure 15:**
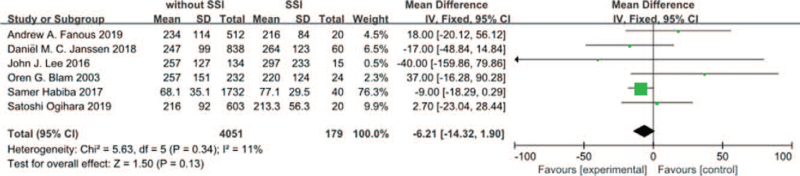
The standardized mean difference (SMD) estimate duration of surgery in 2 groups.

Six studies^[11,14,19,22,24,30]^ reported duration of surgery between SSI group and non-SSI group. There was not significant in the test for heterogeneity and the studies had low heterogeneity (*P* for heterogeneity = .34; *I*^2^ = 11%, Fig. 15). The meta-analysis showed that duration of surgery was not associated with a significant increase in the incidence of SSI (fixed-effects model; *P* = .13, SMD = –6.21, 95% CI [–14.32, 1.90], Fig. 15).

#### 3.3.15 Blood loss

**Figure 16:**
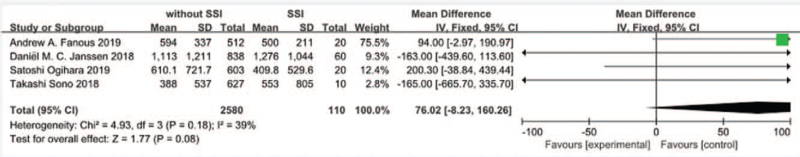
The standardized mean difference (SMD) estimate blood loss in 2 groups.

Four studies^[11,19,21,30]^ reported blood loss between SSI group and non-SSI group. There was not significant in the test for heterogeneity and the studies had low heterogeneity (*P* for heterogeneity = .18; *I*^2^ = 39%, Fig. 16). The meta-analysis showed that blood loss was not associated with a significant increase in the incidence of SSI (fixed-effects model; *P* = .08, SMD = 76.02, 95% CI [–8.23, 160.26], Fig. 16).

#### 3.3.16 Number of fusion level

**Figure 17:**
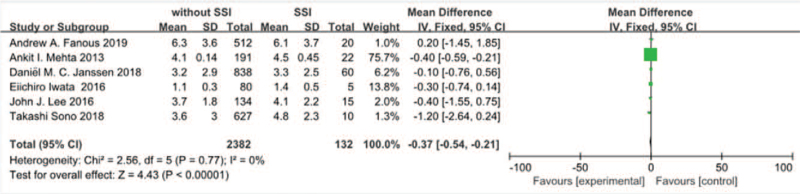
The standardized mean difference (SMD) estimate number of fusion level in 2 groups.

Six studies^[10,11,14,21,30,31]^ reported the number of fusion level between SSI group and non-SSI group. There was not significant in the test for heterogeneity and the studies had low heterogeneity (*P* for heterogeneity = .77; *I*^2^ = 0%, Fig. 17). The meta-analysis showed that the number of fusion level was associated with a significant increase in the incidence of SSI (fixed-effects model; *P* < *.00001*, SMD = –0.37, 95% CI [–0.54, –0.21], Fig. 17).

#### 3.3.17 Surgical location (cervical, thoracic, lumbar)

**Figure 18:**
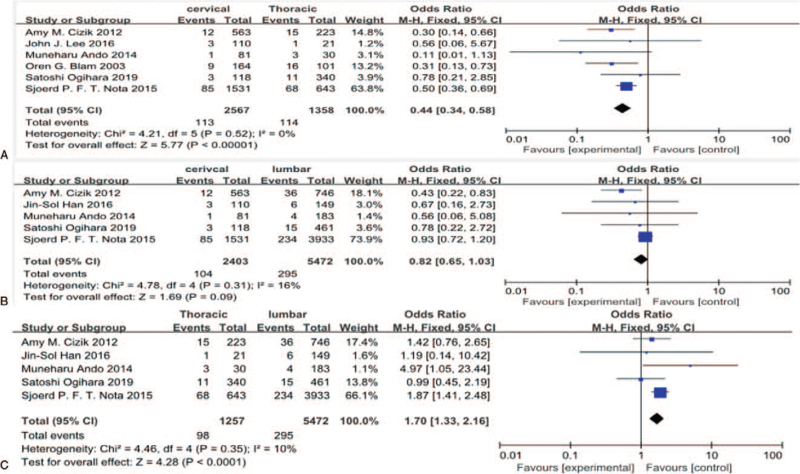
A. The odds ratio (OR) estimate for surgical location (cervical vs thoracic, lumbar). B. The OR estimate for surgical location (cervical vs lumbar). C. The OR estimate for surgical location (thoracic vs lumbar).

Six studies^[11,14,21,30,31,35]^ reported surgical location (cervical, thoracic, lumbar) between SSI group and non-SSI group. There was not significant in the test for heterogeneity and the studies had low heterogeneity (3 *P* for heterogeneity = .52, .31, .35, respectively; *I*^2^ = 0%, 16%, 10%, respectively, Fig. 18). The meta-analysis showed that surgical location (cervical vs thoracic; thoracic vs lumbar) was associated with a significant increase in the incidence of SSI (fixed-effects model; *P* < .0001,  < .0001; OR = 0.44,95% CI[0.34,0.58]; OR = 1.70,95% CI[1.33,2.16], respectively, Fig. 18). However, the meta-analysis showed that surgical location (cervical vs lumbar) was not associated with a significant increase in the incidence of SSI (fixed-effects model; *P* = .09; OR = 0.82, 95% CI[0.65,1.03], Fig. 18).

#### 3.3.18 Fusion approach (anterior, posterior, combined)

**Figure 19:**
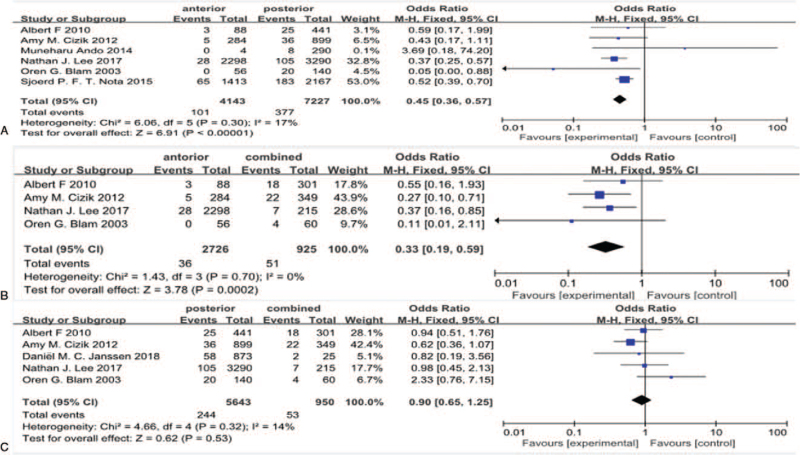
A. The odds ratio (OR) estimate for fusion approach (anterior vs posterior). B. The OR estimate for fusion approach (anterior vs combined). C. The OR estimate for fusion approach (posterior vs combined).

Six studies^[14,19,20,24,26,35]^ reported fusion approach (anterior, posterior, combined) between SSI group and non-SSI group. There was not significant in the test for heterogeneity and the studies had low heterogeneity (3 *P* for heterogeneity = .30, .70, .32, respectively; I^2^ = 17%, 0%, 14%, respectively, Fig. 19). The meta-analysis showed that fusion approach (anterior vs posterior, anterior vs combined) was associated with a significant increase in the incidence of SSI (fixed-effects model; *P* < .0001,  = .0002, respectively; OR = 0.45, 95%CI[0.36,0.57]; OR = 0.33, 95% CI[0.19,0.59], respectively, Fig. 19). However, the meta-analysis showed that fusion approach (posterior vs combined) was not associated with a significant increase in the incidence of SSI (fixed-effects model; *P* = .53; OR = 0.90, 95% CI[0.65,1.25], Fig. 19).

#### 3.3.19 Publication bias

After a detection of publication bias by STATA 12.0, there was no publication bias found for all included studies (all *P* > .05).

## 4 Discussion

SSI is a common disease after spinal surgery in clinic, which may bring great burden on individuals and society. Thus, it is important to find risk factors for SSI after spinal surgery in order to minimize risk as far as possible. Patient characteristics including age, obesity, diabetes, presence of more than 3 co-morbid diseases, urinary incontinence, tobacco use, poor nutritional status, nonsteroidal anti-inflammatory drugs use and surgical factors containing revision surgery, posterior surgical approach, tumor resection, increased estimated blood loss, prolonged surgical time and multilevel surgery fusions extending to the sacrum have been identified as risk factors for SSI in previous studies.^[7,36,37]^ However, the risk factors remain debated due to relatively small numbers of patients.

Previous meta-analysis just have been studied epidemiological incidence of SSI after spinal surgery. As for as we known, there was few meta-analysis regarding few risk factors of SSI after spinal surgery. Thus, we perform a meta-analysis to evaluate the risk factors associated with SSI.^[10–35]^ The incidence of SSI was 2.9% (1222 of 41,624) in our study. Our data also showed that fusion approach (anterior vs posterior; anterior vs combined), osteotomy, transfusion, a history of diabetes, previous surgery, and hypertension, surgical location (cervical vs thoracic; lumbar vs thoracic), osteoporosis and the number of levels fused were associated with development of SSI. However, age, sex, a history of smoking, BMI, fusion approach (posterior vs combined) were, surgical location (cervical vs lumbar), duration of surgery, blood loss, using steroid, dural tear and albumin not associated with development of SSI.

Qi^[16]^ discovered that there was a close relation between diabetes and SSI. Actually, our result was consistent with that of Qi. Patients with diabetes may have lesions in the small vessels and the microvasculature.^[38]^ Therefore, when the vessels are cut, large vessels and microvessels may be occluded, leading to ischemia and hypoxia in the incision tissue and, finally, to infection or a lack of healing at the surgical site. Although many articles found negative correlation between hypertension and SSI, both Amy M. Cizik^[35]^ and Nathan J. Lee^[25]^ reported that hypertension was related with SSI. In the present study, our results implied that hypertension was considered as a risk for SSI after spinal surgery. But we do not understand the reason. Qi Lai^[16]^ was the first to discover the close relation between osteoporosis and SSI following lumbar surgery, which the same with our results. However, the mechanism of osteoporosis and SSI is needed to explore in further study.

Albert F^[29]^ did not find significant relation between the previous surgery and SSI and he believed old scar tissue was not responsible for the increased risk for SSI. However, Cindy R. Nahhas^[13]^ found that reoperation was significantly associated with wound complication. In our study, previous surgery was found to be a risk of SSI after spinal surgery. As for the reason, we inferred that it may be relation to reduction of white blood cell in tissue of scar. Both Nathan^[13]^ and Sjoerd P^[20]^ showed that operative procedure with osteotomy was association with SSI after spinal surgery, which was the same with our finding. As we known, it need more surgical time to perform osteotomy, which markedly increase the incidence of infection due to more time exposure in the air and even transfusion to perform osteotomy. In term of transfusion, no significantly relation was found by Daniël M.^[30]^ While Cindy R. Nahhas^[13]^ and Can Yaldiz^[34]^ demonstrated that transfusion could markedly increase the risk of SSI after spinal surgery. Studies have reported that the immunosuppressive effects of perioperative transfusion may increase the risk of infection at least 2-fold. Regarding the number of fusion level, we proved that it was a risk of SSI. It was easily understood that we spent more operative time and even patients had more blood loss and need more transfusion when we performed more fusion level, which would significantly increase the risk of SSI.

Recently studies tried to compare the rate of SSI for anterior vs posterior spinal surgery and many of these studies offer conflicting views. Pradhan^[39]^ indicated that there was no statistical significance between surgical approaches to fusion. However, Memtsoudis obtained an opposite result by reviewing 261,356 patients and demonstrated that anterior and anterior-posterior fusions were significantly associated with higher rates of complications than posterior fusions. In this meta-analysis, fusion approach (anterior vs posterior, anterior vs combined) was proved to be associated with a significant increase in the incidence of SSI, whereas fusion approach (posterior vs combined) was not associated with it. Anterior approach have a great merit in term of increasing surface area available for fusion and avoiding damage to the posterior supporting muscles. Additionally, a dead space caused by muscle dissection following posterior spinal fusion may predisposes patients to infection more than anterior fusion.^[39–41]^

Jin-Sol Han^[28]^ did not find significant relation between surgical location (cervical, thoracic, and lumbar) and SSI after spinal surgery. While Amy M. Cizik^[35]^ demonstrated that surgical location (cervical, thoracic, and lumbar) was closely association with infection after spinal surgery. Our finding presented that surgical location (cervical vs thoracic and thoracic vs lumbar) was associated with a significant increase in the incidence of SSI, but surgical location (cervical vs lumbar) was not risk for SSI. There is a possible reason may explain these results. We need more surgical time to perform thoracic surgery because it is more risky and difficult than cervical and lumbar surgery, which is easier to cause infection due to exposure from the air for more time.

Many factors including age, history of smoking, BMI, operative time, and blood loss, were not statistically associated with SSI in the present analysis. Especially, in our study, a history of smoking (*P* = .07) and blood loss (*P* = .08) had a negative correlation with SSI. However, we still clinically regard these as potential risk factors of SSI.

There were several limitations in this study. First, we just evaluated SSI totally, which includes superficial and deep SSI. We would discuss superficial and deep SSI respectively in the further study. Second, some factors had 2 included studies. Mentioned above might impact the accuracy of results. Third, some factors, like C-reactive protein (CRP) or other laboratory index, might be risk factors for SSI. Because related studies were few and could not get pooled result, we excluded them.

In conclusion, fusion approach (anterior vs posterior and anterior vs combined), osteotomy, transfusion, diabetes, previous surgery, hypertension, surgical location (cervical vs thoracic and lumbar vs thoracic), osteoporosis and the number of levels fused were associated with a significant increase in the incidence of SSI. In this meta-analysis, we can clearly see which kind of people more likely had SSI after surgery. This article not only provides a reference for spinal surgeons, but also shares decision-making and communication with patients undergoing spinal surgery because some of these factors, such as diabetes, can be adapted during workup. Meanwhile it is helpful for the future study on SSI. Further large-scale, well-designed studies are urgently needed.

## Author contributions

**Software:** Xinxin Zhang.

**Writing – original draft:** Peng Liu.

**Writing – review & editing:** Jipeng You.
